# Deletions in the 17q chromosomal region and their influence on the clonal cytogenetic evolution of recurrent meningiomas

**DOI:** 10.1186/s13039-019-0434-4

**Published:** 2019-05-24

**Authors:** Sina Hemmer, Steffi Urbschat, Joachim Oertel, Ralf Ketter

**Affiliations:** Department of Neurosurgery, Saarland University Hospital, Kirrberger Straße, 66421 Homburg/Saar, Germany

**Keywords:** Recurrent meningioma, Chromosome 17, Genetic alterations

## Abstract

**Objective:**

Meningiomas are among the most frequent intracranial tumors. Although the majority of meningiomas can be cured by surgical resection, up to 20% of the patients develop an aggressive clinical course with tumor recurrence or progressive disease.

Cytogenetically, meningiomas frequently harbour a normal karyotype or monosomy of chromosome 22 as the sole anomaly. However, progression of meningiomas is associated with a non-random pattern of secondary losses of the chromosomes and chromosomal regions 1p, 6, 10, 14, 18, and 19. There is evidence, that loss of chromosome 17 might be involved in the clonal cytogenetic evolution of recurrent meningiomas. The aim of this study was to determine the role of deletions in the 17q chromosomal region in patients with recurrent meningiomas.

**Results:**

The authors retrospectively reviewed all patients that underwent repeated surgery for recurrent meningiomas between 1999 and 2015 at the Department of Neurosurgery of the Saarland University Hospital. Patients were included in this study if tumor samples from two or more different meningiomas were available.

A total of 7 patients underwent repeated surgery for recurrent meningiomas (4 males, 3 females, mean age: 45.4 years at the date of surgery) between 1999 and 2015. Collectively, 22 biopsies were analyzed with FISH (fluorescence-in-situ-hybridization) for the chromosomal region 17q23.3. In 20/22 (90.1%) specimens, the tumor samples harboured a significant deletion in the chromosomal region 17q (range: 10 to 63% of the cells). In 3/3 (100%) cases, deletion in the 17q chromosomal region was detectable in the primary tumor. In the tumor evolution, there was no steady in- or decrease in the percentage of this deletion.

**Conclusion:**

Deletion in the 17q chromosomal region was present in the patients’ primary tumors as well as in late recurrences. Overall, a significant deletion in the 17q chromosomal region was detected in 90.1% of the tumors. Thus, the authors assume that deletion in the 17q chromosomal region displays rather an early event in meningioma progression. Accordingly, deletion in the 17q chromosomal region might clinically serve as a potential early marker for malignancy and a higher risk for recurrence in meningiomas.

## Introduction

Meningiomas are the most frequent intracranial tumors. They account for about 30% of all brain tumors with an annual incidence rate of 10.5 cases per 100,000 females and 4.8 cases per 100,000 males [1]. They are among the cytogenetically best studied tumors and typically reveal a normal karyotype or losses – mostly monosomy and only rarely deletions - of chromosome 22. It is well known that monosomy 22 is not associated with tumor progression or recurrence in meningiomas. However, secondary losses of other chromosomes are accompanied by a more aggressive tumor behavior (rapid tumor growth, infiltrative character) and/or a higher risk of recurrence. Increasing hypodiploidy and complex aberrations are known to induce higher tumor malignancy in meningiomas. Relevant autosomes encompass chromosomes 1, 6, 9, 10, 14, 18 and 19 [2, 3]. As described in the literature, meningiomas with a complex karyotype show higher rates of malignancy [4–6]. Beyond that, epigenetic markers are described to influence meningioma progression and recurrence [7]. However, the current WHO classification of meningiomas subdivides the tumors in three groups based on histological criteria alone [1]. Malignancy and the risk for tumor recurrence increase with higher WHO grading. Recurrence rates after resection Simpson grade I or II are 3–7% for benign, 34.6–38% for atypical and 72.7–78% for anaplastic meningiomas [8, 9]. Valuable histological predictors for meningioma recurrence in benign meningiomas have not yet been identified. To date, loss of the chromosomal region 1p and mutations in *SMO, AKT1* and the *TERT* promoter are independent (cyto-)genetic predictors for meningioma recurrence [3, 7, 10–12].

While aberrations of the chromosomes 1, 10 and 22 and their influence on meningioma recurrence are well studied, aberrations of chromosome 17 are seldomly described.

There are several tumor suppressor genes on chromosome 17 that have been investigated concerning their influence on meningioma progression *(*Table 1*)*.

**Table 1:** Candidate genes on chromosome 17

Gen	Lokus	Funktion
TP53(p53)	17p13.1	Apoptosis, cell cycle control
NF1	17q11.2	Ras signaling, cell cycle control
RPS6KB1	17q23.2	Proliferation control
BRCA1	17q21.31	DNA repair
NM23	17q21.33	Nucleosiddiphosphatkinase

Deletions of *TP53* are associated with higher malignancy and a higher risk for recurrence [13]. A relationship between alterations of *BRCA1* and *BRCA2* and tumor progression in meningiomas could not be proved [14].

*NM23* is a candidate gene on the 17q chromosomal region. Increased expression of *NM23* is associated with a better prognosis in colorectal cancer and breast cancer, whereas deletion in the 17q chromosomal region predicts occurrence of liver metastasis in colorectal cancer [15].

Furthermore, meningiomas with gains of the 17q chromosomal region are also known to show a more aggressive behavior and a higher recurrence rate than meningiomas without this aberration. If there is an increased expression of the oncogene *ERBB2* on the chromosomal region 17q, a shorter progression free survival in colorectal cancer is described [15, 16].

Using comperative genomic hybridization, losses on chromosomes 1p, 6q, 9p, 10, 14q, 18q and 22q as well as gains of genetic material at 1p, 9p, 12q, 15q 20q and 17p were found in anaplastic meningiomas [17–19]. The increase in copy numbers on the long arm of chromosome 17, determined by CGH in 42% of the anaplastic meningiomas investigated by Büschges et al. was substantial, suggesting amplification of DNA sequences.

Also, further studies using non-radioactive in situ hybridization revealed trisomy 17 in meningiomas combined with intratumorous heterogeneity in these cases [20, 21].

While amplification of regions on chromosome 17 is well described in the progression and recurrence of meningioma, there knowledge about the loss of one chromosome 17 in meningiomas and its meaning concerning recurrences and progression in meningiomas is sparse. Here, the authors seek to elucidate the role of deletion in the 17q chromosomal region in recurrent meningiomas.

## Material and methods

### Patient population

The authors retrospectively reviewed all patients that underwent repeated surgery for recurrent meningiomas between 1999 and 2015 at the Department of Neurosurgery of the Saarland University Hospital. Patients were included in this study if tumor samples from two or more different meningiomas were available. *(*Table 2*).*

**Table 2:** Patient population

Patient	Age at first surgery (years)	Date of surgery (m/y)	Tumor	Tumor status
1	46	08/2002	1.1	Recurrence 1
		10/2004	1.2	Recurrence 2
		09/2005	1.3	Recurrence 3
		08/2006	1.4	Recurrence 4
2	38	11/2009	2.1	Primary tumor
		03/2012	2.2	Recurrence 1
		10/2015	2.3	Recurrence 5
3	34	02/2002	3.1	Recurrence 2
		08/2003	3.2	Recurrence 3
		02/2005	3.3	Recurrence 4
4	45	20/2012	4.1	Recurrence 5
		07/2013	4.2	Recurrence 6
		04/2014	4.3	Recurrence 7
		03/2015	4.4	Recurrence 8
5	32	07/1999	5.1	Primary tumor
		01/2002	5.2	Recurrence 2
6	61	03/2001	6.1	Primary tumor
		08/2001	6.2	Recurrence 1
		10/2001	6.3	Recurrence 2
7	62	11/2001	7.1	Recurrence 3
		10/2013	7.2	Recurrence 4

### Fluorescence in-situ hybridization analysis

**Fig. 1:**
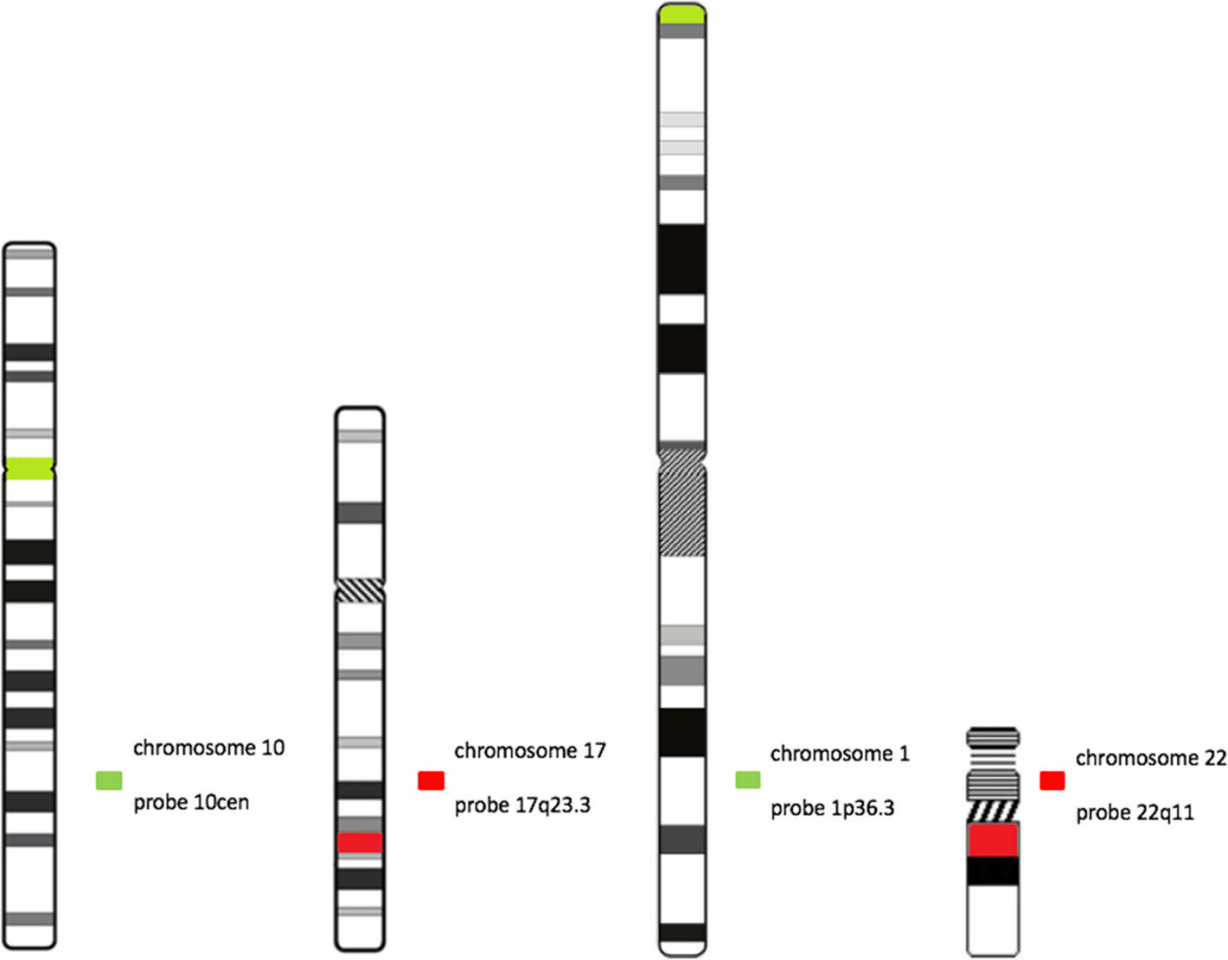
FISH probes for chromosomes 1, 22, 10 and 17 used in this study. The chromosomal regions are described and coloured according to the probes’ fluorescence signal

The tumor samples were examined with direct and indirect fluorescence in-situ hybridization (FISH) for the chromosomal regions 17q, 10cen, 1p and 22q *(*Fig. 1*).* At least two different meningiomas of each patient were examined to evaluate the clonal cytogenetic evolution of recurrent meningiomas. FISH was performed on freshly removed tumor specimens, dapped on silanized microscope slides. FISH after a modified protocol of Pinkel et al. for the chromosomes 1p, 10, 17q and 22q was performed [22]. For chromosome 10, DNA-probes established by our laboratory were used. For the chromosomes 1 and 22, DNA probes by *MetaSystems* were used. For chromosome 17, a DNA probe by *Abott Laboratories* was used. For detection of aberrations of chromosome 10 and the 17q chromosomal region, indirect FISH was performed. Aberrations of the chromosomal regions 1p and 22q were detected by direct FISH.

Samples were analyzed with an *Olympus BX43* fluorescence microscope. At least 200 nuclei of each tumor were analyzed according to the criteria of Hopman et al. [23]. A cut-off value of 10% for alterations of all chromosomes was determined.

#### Chromosomal aberration patterns

Based on the FISH results, chromosomal aberration patterns for each tumor were established. Aberration patterns with chromosomal aberrations in three or more chromosomes within a given tumor were rated as complex patterns.

## Results

FISH analysis was performed on 22 meningioma biopsies (1 WHO I-, 13 WHO II-, 8 WHO III-Tumors) out of 7 patients (4 males, 3 females). Among the 22 biopsies, there were three biopsies of primary tumors and 19 biopsies of recurrences The mean patient age at the first date of surgery was 45.4 years *(*Table 2*).*

### FISH results for chromosome 1p, 10, 17 and 22q

**Fig. 2:**
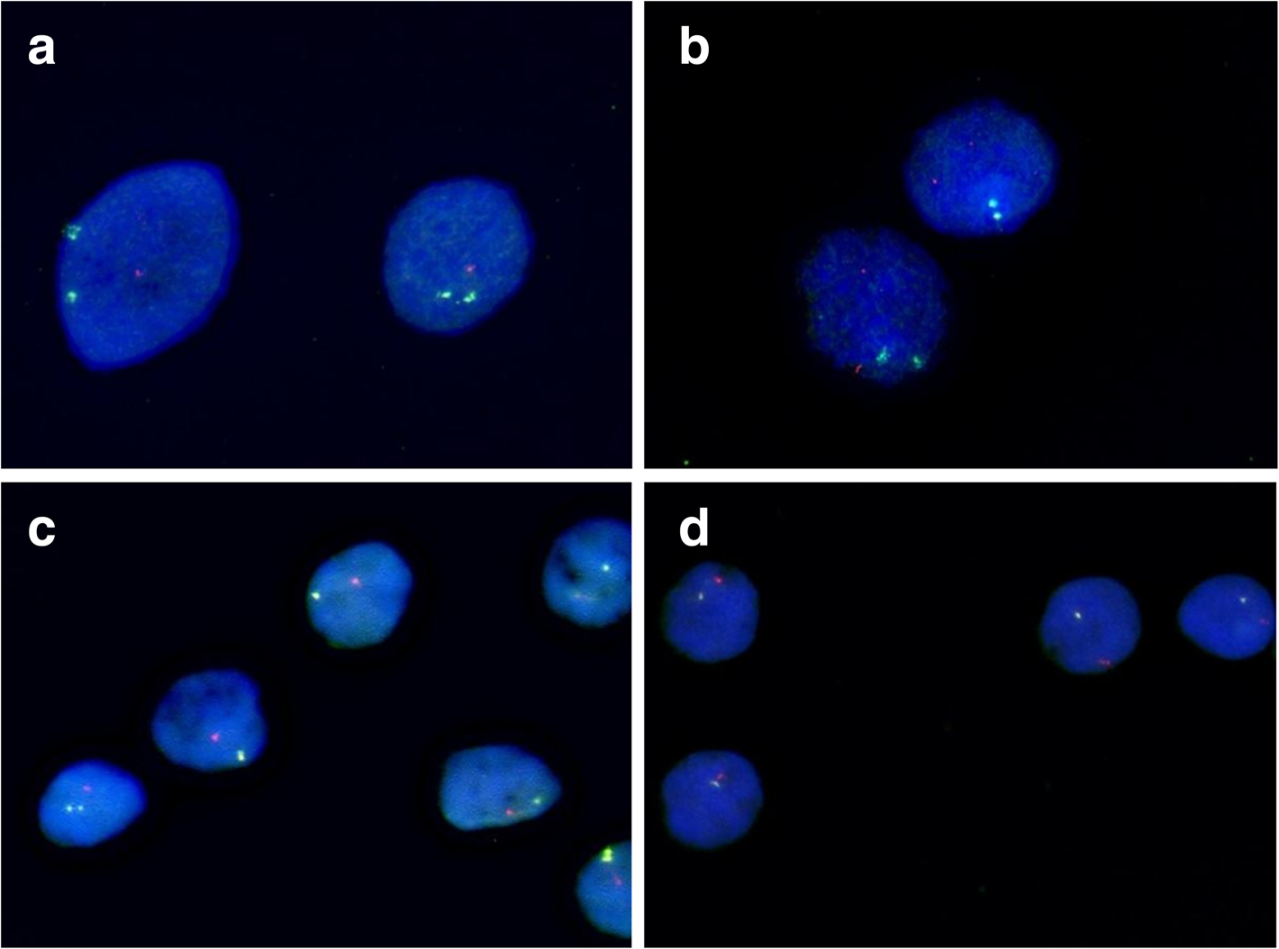
**a** Tumor 6.2, FISH for chromosomes 10 and 17q, deletion of chromosome 17q; green: chromosome 10, red: chromosome 17q. **b** Tumor 6.2, FISH for chromosomes 10 and 17q, deletion of chromosome 17q; green: chromosome 10, red: chromosome 17q. **c** Tumor 1.1, FISH for chromosomes 1p and 22q, loss of chromosomes 1p and 22q; green: chromosome 1p, red: chromosome 22q. **d** Tumor 1.1, FISH for chromosomes 1p and 22q, loss of chromosomes 1p and 22q; green: chromosome 1p, red: chromosome 22q

FISH for the chromosomal regions 1p, 10cen, 17q and 22q was performed on 22 meningioma tissue samples from 7 patients. To evaluate the influence of deletions in the 17q chromosomal region on tumor progression in meningiomas in the course of time, at least two different meningiomas from each patient were analyzed. In 20/22 (90.1%) cases, tumor samples showed a significant deletion in the 17q chromosomal region *(*Figs. 2*-d).* Additional aberrations appeared in the following frequencies: deletion of the chromosomal region 1p in 20/21 tumors (47.6%), deletoin of chromosome 10cen in 6/21 tumors (28.6%), deletion of the chromosomal region 22q in 17/21 tumors (80.9%) and gain of the chromosomal region 17q in 8/21 tumors (38.1%). Table 3 depicts the FISH results as an aberration pattern for each tumor *(*Table 3*).*

**Table 3:** Aberration-patterns for chromosomal regions 10cen, 17q, 1p and 22q. 1: deletion detectable, 0: no deletion detectable; (P) = primary tumor, (R) = recurrence

Patient	Tumor	WHO	-17q	−22q	-1p	-10cen	+17q
Patient 1	1.1 (R)	2	1	1	1	0	0
	1.2 (R)	2	1	1	1	0	0
	1.3 (R)	2	1	1	1	0	0
	1.4 (R)	2	1	0	0	0	1
Patient 2	2.1 (P)	3	1	1	1	0	1
	2.2 (R)	3	1	1	0	1	0
	2.3 (R)	3	1	1	0	0	0
Patient 3	3.1 (R)	2	1	0	0	0	0
	3.2 (R)	2	1	0	0	0	1
	3.3 (R)	2	1	1	0	1	0
Patient 4	4.1 (R)	2	1	1	1	1	0
	4.2 (R)	2	1	1	1	1	0
	4.3 (R)	2	1	1	1	1	0
	4.4 (R)	2	1	0	0	1	0
Patient 5	5.1 (P)	2	1	1	0	0	1
	5.2 (R)	2	1	1	0	0	0
Patient 6	6.1 (P)	3	1	1	1	0	1
	6.2 (R)	3	1	1	1	0	1
	6.3 (R)	3	0	1	0	0	1
Patient 7	7.1 (R)	1	1	1	1	0	0
	7.2 (R)	3	1	1	0	0	0
Frequency total			20/21	17/21	10/21	6/21	8/21
Frequency percentage			95.2%	80.9%	47.6%	28.6%	38.1%

### Chromosomal aberration patterns

**Fig. 3:**
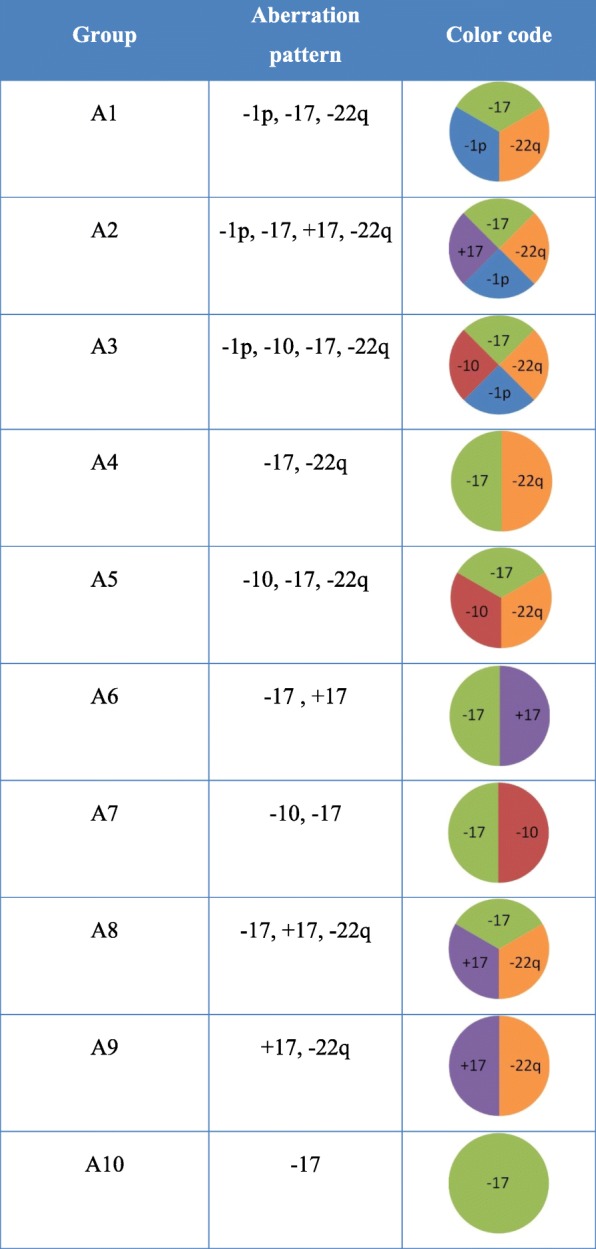
In our study we detected ten different aberration-groups (A1 –A10). We defined the groups A1, A2 and A3 as the groups with the highest mode of aberrations, whereas group A10 represents the group with the lowest complexity of aberrations detected in our study. The tumors for each group are listed according to their specific aberration pattern

**Fig. 4:**
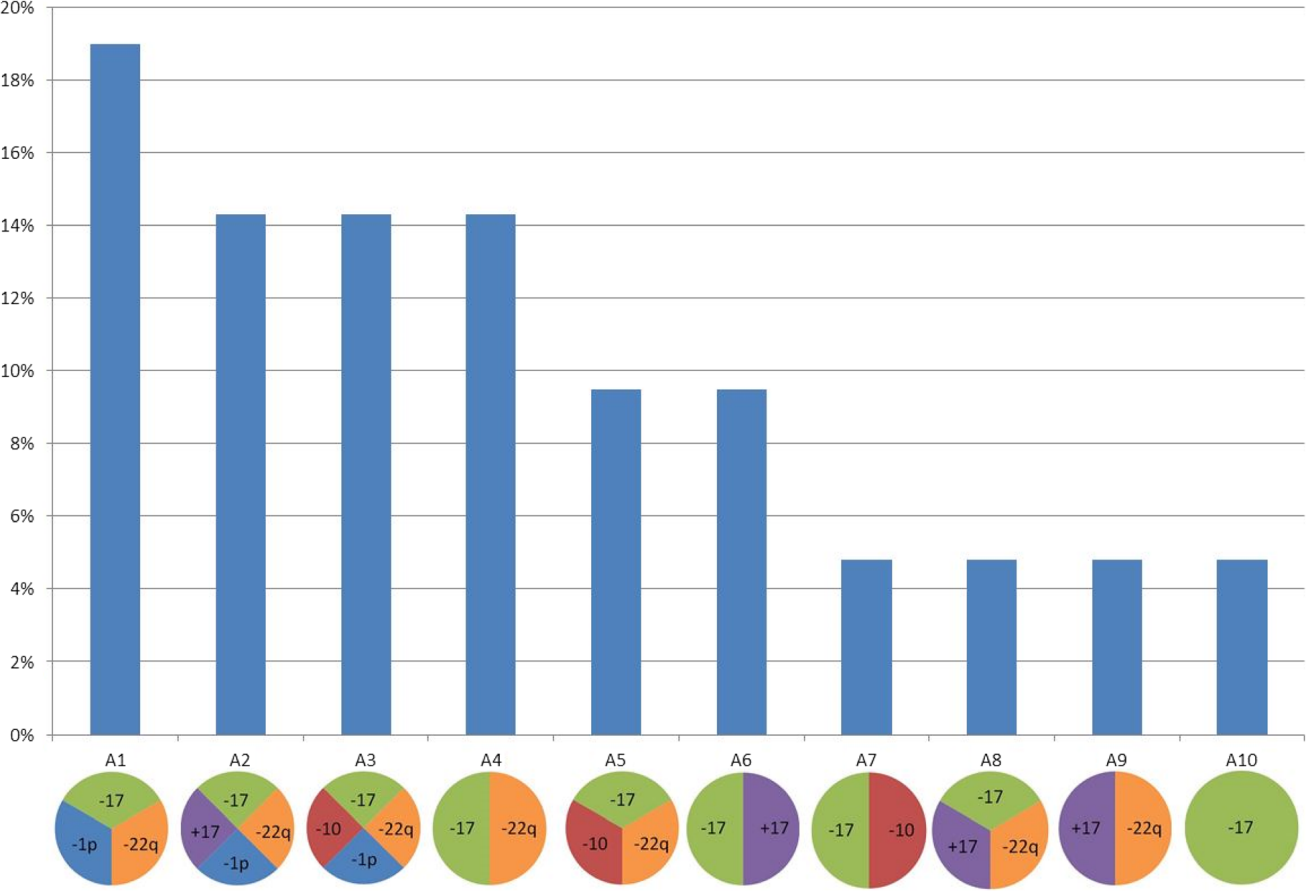
Frequency of the aberration-groups detected in the examined meningiomas

A subdivision of the tumors into aberration groups was conducted to separate simple from complex aberration patterns *(*Fig. 3*).* In our study we detected ten different aberration groups (A1 –A10). Aberration patterns with chromosomal aberrations in three or more chromosomes within a given tumor were rated as complex patterns. The complex aberration patterns of the groups A1, A2 and A3 in which additional loss of one short arm of one chromosome 1 was detectable, accounted for 47.6% of all tumors in this study *(*Fig. 4*).*

### Time course of deletions in the 17 q chromosomal region

Deletions in the 17q chromosomal region were present also in the patients’ early tumor specimens. In 3 cases, specimens of the primary tumor were available. In 3/3 cases (100%), deletions in the 17q chromosomal region were detected in the primary tumor (patients 2, 5 and 6). To evaluate a time-course of deletions of chromosome 17q, at least two meningiomas of each patient were examined. No overall increase or decrease was found in the amount of deletions in the 17q chromosomal region over time.

### Time course of patient 4

Analysis for patient 4 first started with the 5th recurrence of this patient. CGH of the 7th recurrence displayed loss of chromosome 17. FISH revealed a deletion on 17q23.3 in all of the examined specimens and not only in the 7th recurrence.

### FISH vs. CGH/karyotyping

Deletions in the 17q chromosomal region were detected by FISH in 20/22 (90.1%) samples. Only 1/10 (10%) CGH and none of the karyotyping results harboured deletions in the 17q chromosomal region. *(*Table 4*).*

**Table 4:** Comparison of the results from conventional karyotyping and FISH for chromosome 17q

Tumor	Karyotype	FISH 17 for chromosome 17q	
		Aberration	Frequency
1.4	der(1)t(1;9)(p12;q21)	-17q	38.5%
3.1	Normal karyotype	-17q	35.5%
3.2	Normal karyotype	-17q	18.5%
4.1	CGH: -1p, − 10, − 22	-17q	68.5%
4.2	CGH: -1p, −10, −22	-17q	37.5%
4.3	CGH: -1p, −10,-17, − 22	-17q	31.5%
5.1	-22q	-17q	44%
5.2	-22	-17q	57%
6.1	-1p, −22	-17q	11%
6.2	-1p, −22	-17q	18%

## Discussion

Meningiomas are the most frequent intracranial tumours. In addition to the common genetic alteratons in *NF2* in sporadic meningiomas, a number of other clinically actionable genetic events have been described in meningiomas over the past 10 years. Despite tumor resection Simpson grade I and histopathological grading as WHO grade I, some meningiomas recur. To date, loss of the chromosomal region 1p and mutations in *SMO, AKT1* and the *TERT-*promoter are known as independent predictors for meningioma recurrence [3, 7, 10–12]. Further genetic and epigenetic markers are described to influence meningioma progression. Meningiomas with mutations of the *TERT*-promoter are associated with shorter time to progression and a higher risk of recurrence [12]. The influence of deletions in the 17q chromosomal region on meningioma recurrence ist not described in the literature yet. Here, the authors seeked to further elucidate the role of deletions in the 17q chromosomal region in recurrent meningiomas.

### Role of chromosome 17 in meningiomas in the literature

Until today, little is known about loss of chromosome 17 in meningiomas while gain and aberrations of chromosome 17 in meningiomas have previously been described by several authors. Gain of chromosome 17 is associated with a more aggressive behavior of the tumors [18, 19], but the cited studies did either not examine more than one tumor of the same patient, or they did not reveal losses of chromosome 17 with the chosen method. In particular, small mosaics and intratumoral heterogeneity remain undetectable by CGH (Comparative genomic hybridization) or other quantitative methods on tumor samples.

Arnoldus et al. could detect loss of chromosome 17 by FISH (Fluorescence in situ hybridization) in 1 of 30 meningiomas. The examined tumor was a recurrent meningioma [20]. Yakut et al. could detect a deletion of TP53, which is located on chromosome 17p, by FISH in 3 of 34 meningiomas. The three patients developed recurrence during the follow up period [13]. LOH (Loss of heterozygosity) of the chromosomal region 17p, on which TP53 resides, has been described by several authors. However, no statement is made about deletion of the chromosomal region 17q. Lamszus et al. performed LOH analysis on five patients with recurrent meningiomas, but did not examine chromosome 17 [21]. Conventional karyotyping by Lopez-Gines et al. on recurrent meningiomas from nine patients did not reveal loss of chromosome 17 [5].

#### FISH vs. CGH/karyotyping

FISH is known to be more sensitive than CGH and karyotyping after cell culture in detecting mosaic chromosomal aberrations [22, 23]. By comparing FISH and CGH/karyotyping results of the tumors in 10/22 cases we could confirm the results of our former study and Nordkamp et al. [22, 23].

#### Chromosomal aberration patterns

Complex aberration patterns in meningiomas are associated with a more aggressive behavior and a higher risk for recurrence [4–6]. The complex aberration patterns A2 and A3 were the most frequent patterns in our cohort and were rated as complex. The frequency of recurrence of the tumors in this study and the amount of complex aberration patterns can confirm the results that are described in the literature [4–6].

#### Is deletion of chromosome 17q a late event in meningioma progression?

Since loss of chromosome 17 in our patients was first detected in the 7th recurrence of patient 4, we assumed that loss of chromosome 17 could be a late event in meningioma progression. However, despite our presumption of being a late event in meningioma progression, deletions in the 17q chromosomal region were also present in the patient’s primary tumors and early recurrences. No overall increase or decrease in the percentage of deletions in the 17q chromosomal region was detectable*.* Therefore, deletions in the 17q chromosomal region seem to be an early event in meningioma tumor progression associated with a higher risk for recurrence even in the absence of morphological signs of malignancy.

As it is well known that meningiomas mostly show complete monosomy of chromosome 22 rather than partial chromosomal losses like the loss of the short arm of chromosome 1, the authors assume that deletions in the 17q chromosomal region display a loss of the long arm of chromosome 17, or even complete monosomy of chromosome 17.

## Conclusion

Deletions in the 17q chromosomal region may represent a potential early marker for meningioma progression and recurrence when detected in a primary meningioma. Further prospective studies including primary tumors of all WHO grades and higher numbers of specimens should be performed to determine the value of loss of chromosome 17 in meningiomas.

The combined approach of histology and genetic patterns in meningiomas represents the future of meningioma diagnostics, providing a possibility to stratify the risk of recurrence and the aggressive behavior of a given meningioma.
